# Tidal Model in light of complexity: contributions to clinical nursing care in mental health

**DOI:** 10.1590/1980-220X-REEUSP-2024-0377en

**Published:** 2025-04-04

**Authors:** Natália Rocha Chagas Comaru, Ana Ruth Macêdo Monteiro, Lucia de Fatima da Silva, Maria Célia de Freitas, Maria Vilani Cavalcante Guedes

**Affiliations:** 1 Universidade Estadual do Ceará, Fortaleza, CE, Brazil.

**Keywords:** Nursing, Mental Health, Mental Health Assistance, Philosophy, Nursing, Nursing Theory

## Abstract

This is a theoretical essay that aims to reflect on the articulations between Phil Barker’s Tidals Model and Edgar Morin’s Complexity Theory, highlighting Tidals Model adequacy for clinical nursing care in mental health according to the psychosocial care model guidelines. The key concepts of Complexity Theory (dialogic, organizational recursion, hologram and tetragram) were used in an articulated manner with Tidals Model, identifying that this framework is aligned with the following guidelines of psychosocial care: respect for human rights, humanized care, promotion of equity, diversification of care strategies, combating stigmas and prejudices, guaranteeing access to services, and offering comprehensive care and multidisciplinary assistance, under an interdisciplinary logic. Tidals Model represents an alternative to traditional care models, with a care proposal aligned with current public health policies. It is suggested that nurses adopt this framework more effectively, expanding its application to the most diverse audiences and contexts of mental healthcare.

## INTRODUCTION

The incorporation of mental health actions into the Brazilian Health System (In Portuguese, *Sistema Único de Saúde* - SUS) represents a government strategy aimed at consolidating the Brazilian Psychiatric Reform, which began in the 1970s and resulted in the gradual closure of asylums in the country. The anti-asylum struggle enabled the reorientation of care practices in care networks, promoting decentralization of care and greater connection of users to healthcare services, with hospitalization restricted to patients for whom extra-hospital resources were ineffective.

To this end, the Psychosocial Care Network (In Portuguese, *Rede de Atenção Psicossocial* - RAPS) was established by Ordinance MO/MoH 3088/2011^(1)^, which offers comprehensive care, in an articulated manner and at various points of care within the scope of SUS, to people suffering from mental health problems and with needs resulting from the harmful use of alcohol and other drugs.

The RAPS has the following guidelines: “respect for human rights, guaranteeing people’s autonomy and freedom; promoting equity, recognizing the social determinants of health; combating stigmas and prejudices; ensuring access to and quality of services, offering comprehensive care and multidisciplinary assistance, under an interdisciplinary logic; humanized care focused on people’s needs; diversification of care strategies; development of activities in the territory, which favor social inclusion with a view to promoting autonomy and the exercise of citizenship; development of harm reduction strategies; emphasis on territorial and community-based services, with the participation and social control of users and their families; organization of services in a regionalized healthcare network, with the establishment of intersectoral actions to guarantee comprehensive care; promotion of continuing education strategies; and development of the logic of care for people with mental disorders and with needs resulting from the use of crack, alcohol and other drugs, with the central axis being the construction of the Singular Therapeutic Project”^(1)^.

As strategic elements in articulation of RAPS, Psychosocial Care Centers stand out, services regulated by Ordinance 336/2002^(2)^, which “offer care to the population, carrying out clinical monitoring and social reintegration of people with mental disorders through access to work, leisure, exercise of civil rights and strengthening of family and community ties”.

In these devices, nurses plays a fundamental role, acting as one of the pillars of the multidisciplinary team, carrying out actions of reception, active listening and education in mental health, individual and collective, and participating in management of services, through the elaboration of care protocols and activities of units, in addition to promoting articulation between user, family and other services of the health network, guaranteeing continuity of care.

This new care model needs to be absorbed by all professionals, and nurses must be prepared to act in this new logic, open to various possibilities, dialoguing with different existing discourses on psychological suffering, coexisting with the objective and the subjective, with reason and emotion, seeking to multiply questions and distance themselves from comfortable limits, with sensitivity to listening to users and incorporating new care technologies for a humanized nursing practice through differentiated and innovative strategies^(3)^.

To provide mental healthcare from this perspective, clinical practice needs to be based on theoretical frameworks that reinforce the psychosocial care model centered on individuals and their complexities, in contrast to the traditional psychiatric care model, guided by actions restricted to medicalization, diagnosis, signs and symptoms.

Nursing theories, specifically, guide nurses’ care practice in their different functions, giving visibility to the philosophical foundations of nursing, and are important tools to support this paradigm shift.

To support and qualify clinical nursing care in mental health, Tidal Model emerged in the mid-1990s, created by Scottish psychiatric nurse Phil Barker, which advocates the existence of cooperative work between nurse and patient, in which professionals take over as apprentice about the experience lived by a person.

Tidal Model emphasizes the need to discover what mental health means to each individual, since its meaning varies from person to person in a unique manner. It considers that everything is marked in people’s life stories, which show all the changes that have occurred during the journey since birth, and how they responded to each of them. For Tidal Model, recovering these personal stories and problems as human beings represents the first step towards regaining control over life^(4)^.

This is a theory that encourages theoretical-conceptual changes, developed specifically for mental health nursing practice, which allows nurses to qualify care for people in distress and, consequently, strengthen the Psychosocial Care Policy in the country^(5)^. It moves away from the conventional paradigm, focused on the figure of specialists, with a reductive pharmacological approach, placing the person at the center of nursing care and allowing them to feel involved in the entire process of recovering their mental health.

Furthermore, it proposes working with and managing “the person at risk”, and not the “risk” itself, redirecting nursing practices towards a path of comprehensiveness and, consequently, with therapeutic responses.

Tidal Model, with its holistic and person-centered approach, represents a significant evolution in relation to classical nursing theories, such as those of Peplau and Travelbee. Although it shares with these theories a focus on the therapeutic relationship and patients’ experience, Tidal Model presents unique characteristics that differentiate it and make it a valuable tool for contemporary mental health nursing practice. While Peplau’s and Travelbee’s theories provided a solid foundation for understanding the nurse-patient relationship and the meaning of illness, Tidal Model offers a broader and more complex perspective that takes into account patients’ social, cultural and historical context, emphasizing the importance of narrative, change and the person’s role.

Tidal Model does not replace classical theories, but complements them from a more comprehensive and updated perspective, offering practical tools for nursing assessment and intervention, allowing nurses to provide more humanized, effective care focused on the person’s needs. Due to this differentiated nature, and considering the multidimensionality of a person in psychological distress, Tidal Model assumptions contain elements that are similar to the complex thinking proposed by Edgar Morin, who affirms the need for a new understanding of the subject that potentially recognizes that every person is a subject, actor and author capable of cognition, choice and decision, in a social environment of intersubjective relationships^(6)^.

Complexity allows for the joint elaboration of distinct or different elements, towards a global understanding of phenomena, providing an understanding of health as a complex phenomenon at individual, social and structural levels^(7)^. The theoretical perspective of complexity helps to rethink the ontology of human beings, nurses and care, and to reformulate nursing knowledge, overcoming the paradigm of simplification and fragmentation, in search of new ways of caring, teaching and managing care^(8)^.

In view of this, there was an interest in reflecting on the articulation between Tidal Model and Complexity Theory, highlighting Tidal Model adequacy for clinical nursing care in mental health according to the psychosocial care model guidelines. With this, we hope to assist nurses in using the nursing discipline’s own framework in mental healthcare, strengthening their professional identity. This is in line with the Federal Nursing Council (In Portuguese, *Conselho Federal de Enfermagem* - COFEN) recommendation, which, through COFEN Resolution 678 of August 19, 2021^(9)^, establishes the actions of generalist nurses in the context of mental health and advocates the use of theoretical models to support and systematize mental healthcare actions. Furthermore, it collaborates in the search for a framework that presents a new conception of mental health and psychological distress in line with deinstitutionalization assumptions.

## METHOD

This is a theoretical reflection with a qualitative approach that emerged from discussions held in the discipline critical analysis of clinical care in nursing and health, offered in a doctoral course of a graduate program in clinical care in nursing and health at the *Universidade Estadual do Ceará*. The key concepts of Edgar Morin’s Complexity Theory were used, intertwined with Phil Barker’s Tidal Model, highlighting the benefits of applying the model in practice of clinical nursing care in mental health, evidencing the consonance of Tidal Model with the psychosocial care model guidelines recommended by the Ministry of Health.

The proposal to articulate Complexity Theory with Tidal Model offers a rich perspective for understanding the challenges and opportunities faced in an increasingly interconnected and complex world. Both theories, although originating from different fields, share the premise that systems, whether social, organizational or individual, are intrinsically complex and cannot be reduced to a simple sum of their parts.

There are several other points of convergence and synergy between the two theories, in addition to emphasis on a systemic vision, highlighting dynamics and change as inherent characteristics of systems and the need to develop more effective approaches to deal with uncertainty and complexity, in a process of continuous learning, which meets the premises of comprehensive care in mental health.

The reading and study of the books “Introduction to Complex thinking”^(10)^, by Edgar Morin, and “*El Modelo Tidal: salud mental, reinvidicación y recuperación*”^(4)^, by Phil Barker and Poppy Buchanan-Barker, in which the authors describe their respective theories, in addition to the reading of other related textual productions, allowed structuring this theoretical reflection, organized into three axes: “The Tidal Model”, which briefly presents Tidal Model assumptions, enabling a general approach to Phil Barker’s thinking; “Considerations on Edgar Morin’s complex thinking”, which in turn also seeks to clarify key concepts of Complexity Theory; and “Tidal Model from the perspective of complexity: implications for clinical nursing care in mental health”, which demonstrates the articulation between the two models, identifying the interrelationship between Tidal Model and the psychosocial care model guidelines, presenting the use of theory as a stimulus for clinical nursing care in mental health from new perspectives.

## THE TIDAL MODEL

Tidal Model is a nursing theory that proposes person- centered care, helping them discover the problems in daily life that are the cause of their psychological distress as well as mobilizing their own resources to respond to them, recovering their mental health.

The theory is internationally recognized and has wide application in diverse contexts, such as outpatient clinics, rehabilitation clinics, home care, among others. Moreover, it allows individual and collective work aimed at various audiences, such as children, adolescents, older adults and families.

A study published in 2022 on the applicability of Tidal Model by nurses in mental healthcare services identifies the following results from the application/incorporation of this theory into nursing care: reduction in physical aggression, violence directed at others or oneself, and harassment; reduction in the use of psychoactive substances; increased resilience of women victims of violence; improvement in a person’s coping strategies and self-esteem; nurse satisfaction; positive assessment by the service user regarding the care provided; broadness of nurses’ assessment related to the health status of a person with suicidal ideation; reduction in length of hospitalization and use of restraint; closer ties between nurse and person in need of care; and valorization of nursing practice in mental health^(5)^.

Phil Barker’s model enriches the theoretical body of nursing, as it redefines the paradigm of mental healthcare, providing opportunities for interprofessional work and guaranteeing autonomy to nurses and individuals with psychological distress in the care process^(11)^. Using the metaphor of water, the theory postulates that human experience is fluid in nature and considers life as an ocean of experiences, in which change is a constant and unlimited process. On this journey of evolution, exploration and discovery, one encounters unpredictable problems that one cannot always deal with. At this point, one experiences an emotional shipwreck, in which public manifestations arise that something is not right, although the experience of distress remains invisible in the “Self” domain. To be closer to the meaning of this personal experience, nurses have the mission of establishing sincere and authentic communication, valuing the person’s expression, their voice, their language so that human concern is their guide, in a feeling of connection (bridging relationship) triggered by means of the key tool of the therapeutic process: the interpersonal relationship.

Tidal Model presents six guiding principles: the virtue of curiosity, which consists of investigating what motivated the search for mental healthcare; the power of resourcefulness, which seeks to reveal how a person lives or coexists with distress/illness; the value of respecting the person’s wishes in a care process based on active collaboration, centered on the person; the paradox, crisis as an opportunity that sees the crisis as an opportunity for change; everyday wisdom, which establishes goals in which small daily steps are outlined to achieve specific objectives; and the search for elegance, in the elaboration of a nursing care plan that contains simple actions so that the person experiences a change focused on what is necessary^(4)^.

The model is based on four fundamental questions: why this, why now? Pay attention to what the person is experiencing at the moment. What works? What has worked or could work to solve the problem from the person’s perspective? What is the person’s “personal theory”? How do they understand and explain what is happening and how can they limit the restrictions? Do as little as possible for the person, and allow them to do it themselves^(4)^.

Tidal Model lists ten commitments that are the essence of its application in professional practice: valuing individuals’ voice, allowing them to express their experience and tell their story; respecting their language, encouraging them to speak in their own words as they understand what they are experiencing; becoming an apprentice, learning from others; using the available “toolkit”, helping the person to identify what “worked” or “what could work” to help them deal with the problems they experience; developing the ability to go one step further, detecting what needs to be done “now” to help move forward; giving the gift of time; developing genuine curiosity, identifying the necessary information; knowing that change is constant; revealing personal wisdom, recognizing the person’s potential in their recovery process; and being transparent^(11)^. Meeting each of these commitments requires specific skills from the nurse that need to be developed to generate a situation of learning from others and co-responsibility for care.

Finally, the continuum of care proposed by Tidal Model guides what type of care a person in psychological distress needs to receive, according to their state and health needs, from immediate care, in the face of a storm or drowning so that the person can reestablish their path, through transitional care, which helps the person to continue their journey, understanding what happened to cause them to deviate from their path, to developmental care, which enables the person to deal with these situations in the future, until they are able to act independently of a healthcare service, resuming their daily activities^(4)^.

## CONSIDERATIONS ON EDGAR MORIN’S COMPLEX THINKING

The word “complexity” comes from the Greek term *complexus*, which can be translated as “that which weaves together”. However, in common sense, it has come to be used as a synonym for “complicated”, “difficult to understand”.

In the so-called complex thinking of French philosopher Edgar Morin, which has been developed and expanded since the 1960s, which sees the world as an inseparable whole, complexity is understood as “the fabric of events, actions, interactions, feedbacks, determinations, coincidences that constitute the phenomenal world, and presents itself with the disturbing features of entanglement, inextricability, disorder, ambiguity, uncertainty”^(10)^.

Edgar Morin uses principles of Information Theory, Cybernetics Theory, Systems Theory, Self-Organization Theory and Microphysics Theory, as well as the concept of open system, which emphasize the subject/object relationship, which contributes to the paradigmatic change that proposes the aggregation of knowledge rather than the fragmentation and reductionism of classical science^(12)^.

In his model, he states that uncertainty and contradictions are part of life and the human condition, and that the reconnection of beings and knowledge is only possible through solidarity and ethics.

Morin’s Complexity Theory aims to exercise a way of thinking that is capable of dealing with, dialoguing with and negotiating reality, prioritizing articulations between disciplines that are dismembered by disjunctive thinking, which isolates what separates and hides what reconnects. To this end, it proposes replacing the dichotomous way of thinking of dualities subject-object, part-whole, reason-emotion, among others, of the Cartesian view with an articulated way of thinking, abandoning the paradigm of a mechanistic universe, understanding the world from the perspective of complex systems.

The theory’s key concepts are: dialogic, which understands phenomena as antagonistic, competing and complementary simultaneously; organizational recursion, which postulates that the effects of a process are also its co-producers (self- organization and self-production); hologram, which states that the part is in the whole, as the whole is in the part; and tetragram of complexity, represented by the order- disorder-interaction-organization paradigm^(10)^. From this perspective, it is possible to maintain the duality present in unity and conceive order and disorder as relative and relational concepts, both cooperating to organize the universe.

Complexity Theory has spread throughout the world, and its principles are widely applied in studies in the fields of education, health and organizations, among others, opening new perspectives for scientific knowledge in general and favoring the creation of innovative approaches to problem-solving.

## TIDAL MODEL FROM THE PERSPECTIVE OF COMPLEXITY: IMPLICATIONS FOR CLINICAL NURSING CARE IN MENTAL HEALTH

Integrating the Complexity Theory precepts, as presented by Edgar Morin, with Phill Barker’s Tidal Model, relating them to the psychosocial care model guidelines, is of utmost importance, as it promotes a conceptual deepening that goes beyond the simple comparison between theories, enriching knowledge in nursing and expanding the understanding of the complex phenomena that permeate mental healthcare.

RAPS, which aspires to promote citizenship and social inclusion of people with mental illness, as well as the comprehensiveness and continuity of their care, is a living example of a paradigmatic and thought reform, supporting Morin’s perspective that hyperspecialization, which fragments the global and dilutes the essential, must give way to complex thinking that is capable of perceiving the interactional process between the parts and the whole and, thus, solving multidimensional problems.

By adopting a complex perspective, nursing professionals are encouraged to transcend reductionist and fragmented models, recognizing the interdependence between the various factors that influence mental illness. This approach allows for a more comprehensive and humanized practice, aligned with the RAPS principles.

RAPS is established in multiple connections and interrelations in the territory. From the perspective of complexity, it is possible to distinguish the objectivity of the laws and ministerial orders that regulate it and the subjectivity of its actors and interaction in services in all their dynamism. In this living tangle, one can perceive order and disorder in constant interaction, which allows the network to materialize, self-organize and advance^(12)^. Tidal Model, in turn, when analyzed from the perspective of Complexity Theory, offers a robust theoretical framework for mental health nursing practice. Considering that mental illness encompasses a wide variety of human life problems, Tidal Model emphasizes that nurses must mobilize the essence of nursing care, which is to provide support to the person, and states that authentic nursing takes place when the person begins their experience of growth and development.

Nurses must be flexible, pragmatic and, above all, creative in mental healthcare, as they are the main therapeutic tools, helping the person to contextualize their health-disease process and identify what works for them, developing their personal theory about what they feel, facilitating, without major interference, the recovery of their mental health, in a movement of constant human appreciation^(13)^. Thus, the ethics of human understanding begins when human beings are conceived and felt as subjects, which makes professionals sensitive to their joys and sufferings, representing a fundamental requirement of current times of widespread misunderstanding^(14)^.

Realizing these similarities between the two theories, we proposed a discussion here about the principles of complexity applied to Tidal Model and its adequacy to RAPS guidelines, which is illustrated in Chart 1 for better visualization.

**Chart 1:** Application of complexity principles in Tidal Model and in the Psychosocial Care Network guidelines – Fortaleza, CE, Brazil, 2025.

Complexity principle	Application in Tidal Model	Psychosocial Care Network guidelines^(1)^
**Dialogic**	– Complementary antagonisms are accepted: health x mental illness; nurse x person in psychological distress; whole x parts (domains of the Self, the World and Others); – In its fourth guiding principle, it views the crisis as an opportunity for change.	– “Respect for human rights, guaranteeing people’s autonomy and freedom”; – “Humanized care focused on people’s needs”; – “Promotion of equity, recognizing the social determinants of health”.
**Organizational recursion**	– Change is a constant process; – Knowledge and discoveries about the person’s relationship with health and illness, constructed and revealed in collaboration with the nurse, help to form new knowledge, which generates new perspectives on the subject.	– “Respect for human rights, guaranteeing people’s autonomy and freedom”; – “Humanized care focused on people’s needs”; – “Diversification of care strategies”.
**Hologram**	– The person is represented and must be understood in three personal domains: the Self, the World and Others.	– “Promoting equity, recognizing the social determinants of health”.
**Tetragram of complexity**	– He considers that the unpredictability of nature is similar to human life, comparing it to the flow of water, the ebb and flow of the tides, drawing his central philosophical metaphor from Chaos Theory. – Life and its unpredictability are threatening, and everyday human problems arise from complex interactions between people and them, enabling the installation of chaos.	– “Combating stigmas and prejudices; ensuring access and quality of services, offering comprehensive care and multidisciplinary assistance, under an interdisciplinary logic”.

Source: prepared by the authors.

Dialogic permeates Tidal Model to the extent that concepts that are essentially antagonistic by definition are exposed in complementarity. As in a continuous connection between opposites, such as health and mental illness, nurse and person in psychological distress, the whole (represented by the person) and the parts (the domains of the Self, the World and Others) appear interconnected, characterizing nursing as an interactive activity related to human development and growth.

Furthermore, Tidal Model’s guiding principle, which highlights the crisis as an opportunity for change, places nurses face to face with the dialogic highlighted by Morin, in which “the being, organizer-of-themselves, in order to organize itself, needs to close itself in relation to the ecosystem, but also be open in relation to it, because it is in this interactive process that it organizes itself, expressing the inseparable relationship between subject and world, i.e., the world is in the subject and the subject in the world”^(14)^. Therefore, permeated by complexity, Tidal Model reaffirms interpersonal relationships as an essential tool for successful mental health nursing care. It is in line with the paradigm of psychosocial care, which recommends assisting people with mental distress primarily in territorial-based services and understanding their way of life and relationships, allowing them to maintain their autonomy and freedom.

Organizational recursion is present in Tidal Model’s main premise, according to which change is constant, and it is up to nurses to identify what type of change represents a step forward in the process of mental health recovery, developing in the person the awareness that even small steps are capable of promoting transformations. At this point, there is a consonance with Edgar Morin’s assumption, according to which all the results of a process are also its co-producers, in a self-constituting and self-organizing circuit that encourages dynamic, interactive and recursive actions, abandoning the linear conception of cause and effect.

The interplay of theories reinforces the idea that the path to mental health recovery is unique. In this way, each individual will find the best path in the flow of life to achieve their well-being, which is in line with humanized care focused on people’s needs, proposing diverse care strategies towards comprehensiveness.

Tidal Model also expresses the need to understand the person as a whole in three domains: the Self, which is the private place of human experiences, known completely only by the person; the World, in which the person shares some experiences of the Self domain with others in the social world; and Others, which is the scenario in which the person shares daily life with family, friends, neighbors, coworkers, professionals, etc., affecting them and being affected by them^(13)^.

This premise supports the holographic principle, which goes beyond reductionism, which only sees the parts, and holism, which only sees the whole, admitting that the part is in the whole, but also that the whole is in the part. It recognizes that it is possible to increase the understanding of the parts by the whole and of the whole by the parts, moving from disjunctive and reductive thinking to complex thinking. Contextualizing, therefore, allows us to attribute meaning to human care, making the necessary skills for caring for others and for life flourish, far beyond cognitive skills, through the understanding of spaces where everything is constructed and reconstructed all the time, in a process permeated by varied emotions^(15)^. Thus, human understanding promotes harmony among different people, whether due to their choices, ethnic origin, sexual orientation or experience of distress, helping to pave the way for equity, considering the social determinants of health, as proposed by the psychosocial care model.

Finally, the tetragrammaton of complexity permeates Tidal Model by stating that only with the help of the order-disorder paradox is it possible to promote organization. The concept of order goes beyond the deterministic idea of stability, relying on the idea of mutual interactions, considering that nothing exists without internal and external influences and their interdependence. Furthermore, disorder abandons the idea of chance, despite always admitting it, because for there to be disorder, there must be “separation, instability and inconstancy”^(14)^. Therefore, Complexity Theory is based on a thought that considers all influences, internal and external, highlighting uncertainty as a guiding principle, and does not propose its elimination; on the contrary, it suggests that it is necessary to understand contradiction and the unpredictable based on the coexistence among them^(14)^.

The integration of these theories favors: nurses’ professional practice, which implies personalization of care, recognizing the uniqueness of each person, their life history and social context; autonomy promotion, encouraging patient participation in their recovery process, valuing their voice and experience; the use of flexibility and creativity to navigate situations of uncertainty and adapt their approaches; the strengthening of the therapeutic relationship, in which nurse-patient interaction becomes central, promoting a bond based on mutual understanding and respect.

Thus, complex thinking and Tidal Model facilitate the development of care actions in uncertain environments, such as in the face of crises, as these actions are produced by concomitant analysis of defined (order) and undefined (disorder) conditions, and it is from this assessment that a complex mental healthcare plan emerges that respects life stories and the meanings of health and illness for people in psychological distress.

The linear, deterministic conception based on certainty needs to be reformulated to create models that contemplate the complexity of life. This implies adopting theoretical principles in tune with the advances of nursing as a science, art and profession. In this regard, complexity represents an approach that emphasizes inter and transdisciplinarity as alternatives to restructuring knowledge in contemporary times^(8)^.

The approach to subjectivity needs to bring nursing closer to essential skills and abilities for humanization of care, since clinical and humanized work in mental health requires time for reception, qualified listening, attention and interpersonal relationships with the subjects who suffer, with a view to reorienting care from the perspective of privileging the possibilities and potentialities of subjects with a view to social reintegration^(16)^.

Complexity Theory is used, in particular, to understand the relationships and phenomena involved in nursing care in its entirety and in different phases of human development, emphasizing the need to incorporate multiple perspectives for its execution^(17)^. Considering nursing practice broadly related to the complexity of Being, nurses, as social beings with technical and scientific knowledge, must reflect on new forms of care that can encompass dynamism and comprehensiveness, articulating care networks and interconnections that depend on their performance^(17)^.

The articulation between Complexity Theory and Tidal Model: allows us to understand phenomena in their complexity, considering the interconnections between the different elements (systemic vision); facilitates adaptation to constantly changing scenarios and the creation of more flexible solutions (adaptability); promotes collaboration between different actors and the construction of joint solutions (collaborative work); and contributes to the construction of more resilient systems and organizations capable of dealing with uncertainty (resilience).

As a practical application to clinical nursing care in mental health, there is: the establishment of more significant interpersonal relationships, understanding the multiplicity of factors involved in the experience of distress of subjects, capable of providing greater connection of users and family members to services and, consequently, greater recognition and professional satisfaction of nurses; the possibility of developing more specific and effective Singular Therapeutic Projects, considering the centrality of the person and their history; and the creation of new individual and collective strategies for multidisciplinary work in an integrated manner, with a broader view, so important to meet the multiple demands presented by people in psychological suffering.

The dialogue between Complexity Theory and Tidal Model allows nurses to develop actions that contribute to the premises of fighting stigmas and prejudices, ensuring access to and quality of services, and offering comprehensive care and multidisciplinary assistance from an interdisciplinary perspective, strengthening the psychosocial care model.

## CONCLUSION

In order to consolidate mental health nursing as a holistic, inclusive, positive and collaborative practice, to the detriment of psychiatric nursing, which is paternalistic, coercive, negative and disease-oriented, it is urgent to offer clinical mental healthcare that goes beyond the biological, dichotomous and fragmented view, guided by the use of medications, in order to understand the person in psychological distress in a multidimensional way, connecting the subject to their sociocultural dimension. To favor this expanded view, Phill Barker’s Tidal Model proposal contains elements that dialogue with Edgar Morin’s Complexity Theory, which meet the psychosocial care model guidelines, thus presenting itself as a theory capable of supporting and directing mental health practice and its complexities.

It is suggested that nurses deepen their study of nursing theories focused on mental healthcare, reflecting critically on them, contributing to improving models, as well as adapting and expanding their application in different contexts as a way of favoring their protagonism as a therapeutic agent, collaborating in the leading role of subjects in psychological distress and achieving the consolidation of the much desired psychosocial care in the health system.

Tidal Model and Complexity Theory, although distinct, converge in their fundamental principles for mental healthcare. By integrating these concepts, a more robust theoretical framework is created that allows us to understand human beings in its entirety, recognizing the complexity inherent in the processes of illness and recovery in mental health. This reflection is crucial to overcome reductionist and fragmented models, promoting a nursing practice that values subjectivity, the interrelationship between the individual and the environment, and the constant transformation present in human life.
