# Supplementary material for: Tidal Model in light of complexity: contributions to clinical nursing care in mental health
Source: Rev Esc Enferm USP. 2025 Apr 4;59:e20240377. doi: 10.1590/1980-220X-REEUSP-2024-0377en (PMC11970829; doi:10.1590/1980-220X-REEUSP-2024-0377en)
Supplement: Translation

# Translation: *Tidal Model* à luz da complexidade: contribuições ao cuidado clínico de enfermagem em saúde mental

**DOI:** 10.1590/1980-220X-REEUSP-2024-0377pt

**Authors:** Natália Rocha Chagas Comaru, Ana Ruth Macêdo Monteiro, Lucia de Fatima da Silva, Maria Célia de Freitas, Maria Vilani Cavalcante Guedes

**Affiliations:** 1 Universidade Estadual do Ceará, Fortaleza, CE, Brasil.

**Keywords:** Enfermagem, Saúde Mental, Assistência à Saúde Mental, Filosofia em Enfermagem, Teoria de Enfermagem

## Abstract

Trata-se de ensaio teórico que objetiva refletir sobre as articulações entre o *Tidal Model* (Modelo das Marés)*,* de Phil Barker, e a Teoria da Complexidade, de Edgar Morin, evidenciando a adequação do *Tidal Model* para o cuidado clínico de enfermagem em saúde mental segundo as diretrizes do modelo de atenção psicossocial. Os conceitos-chave da Teoria da Complexidade (dialógica, recursividade organizacional, holograma e tetragrama) foram utilizados de forma articulada com o *Tidal Model*, identificando que este referencial está alinhado às seguintes diretrizes da atenção psicossocial: respeito aos direitos humanos, atenção humanizada, promoção da equidade, diversificação das estratégias de cuidado, combate a estigmas e preconceitos, garantia de acesso aos serviços, e oferta de cuidado integral e assistência multiprofissional, sob a lógica interdisciplinar. O *Tidal Model* representa uma alternativa de superação aos modelos tradicionais de assistência, com uma proposta de cuidado alinhada às atuais políticas públicas de saúde. Sugere-se maior apropriação desse referencial pelos enfermeiros, expandindo sua aplicação aos mais diversos públicos e contextos de cuidado em saúde mental.

## INTRODUÇÃO

A incorporação de ações de saúde mental no Sistema Único de Saúde (SUS) representa uma estratégia governamental com vistas à consolidação da Reforma Psiquiátrica Brasileira, iniciada na década de 1970, que resultou no fechamento gradual de manicômios no país. A luta antimanicomial possibilitou a reorientação da prática assistencial em redes de atenção, promovendo a descentralização do cuidado e uma maior vinculação dos usuários aos serviços de saúde, ficando a internação restrita a pacientes para os quais os recursos extra-hospitalares se mostram ineficazes.

Para tanto, foi instituída, pela portaria GM/MS 3088/2011^(1)^, a Rede de Atenção Psicossocial (RAPS), que oferece atendimento integral, de forma articulada e em vários pontos de atenção no âmbito do SUS, a pessoas em sofrimento psíquico e com necessidades decorrentes do uso nocivo de álcool e outras drogas.

A RAPS tem como diretrizes: “o respeito aos direitos humanos, garantindo a autonomia e a liberdade das pessoas; a promoção da equidade, reconhecendo os determinantes sociais da saúde; o combate a estigmas e preconceitos; a garantia do acesso e da qualidade dos serviços, ofertando cuidado integral e assistência multiprofissional, sob a lógica interdisciplinar; a atenção humanizada e centrada nas necessidades das pessoas; a diversificação das estratégias de cuidado; o desenvolvimento de atividades no território, que favoreça a inclusão social com vistas à promoção de autonomia e ao exercício da cidadania; o desenvolvimento de estratégias de Redução de Danos; a ênfase em serviços de base territorial e comunitária, com participação e controle social dos usuários e de seus familiares; a organização dos serviços em rede de atenção à saúde regionalizada, com estabelecimento de ações intersetoriais para garantir a integralidade do cuidado; a promoção de estratégias de educação permanente; e o desenvolvimento da lógica do cuidado para pessoas com transtornos mentais e com necessidades decorrentes do uso de crack, álcool e outras drogas, tendo como eixo central a construção do Projeto Terapêutico Singular”^(1)^.

Como elementos estratégicos na articulação da RAPS, destacam-se os Centros de Atenção Psicossocial, serviços regulamentados pela Portaria n° 336/2002^(2)^, que “oferecem atendimento à população, realizando o acompanhamento clínico e a reinserção social de pessoas com transtornos mentais pelo acesso ao trabalho, lazer, exercício dos direitos civis e fortalecimento dos laços familiares e comunitários”.

Nesses dispositivos, o enfermeiro desempenha um papel fundamental, atuando como um dos pilares da equipe multidisciplinar, realizando ações de acolhimento, escuta ativa e educação em saúde mental, individual e coletiva, e participando da gestão dos serviços, mediante a elaboração de protocolos de atendimento e atividades das unidades, além de promover a articulação entre usuário, família e outros serviços da rede de saúde, garantindo a continuidade do cuidado.

Esse novo modelo de atenção precisa ser absorvido por todos os profissionais, e o enfermeiro deve estar preparado para atuar nessa nova lógica, aberto a várias possibilidades, dialogando com diversos discursos existentes sobre o sofrimento psíquico, convivendo com o objetivo e o subjetivo, com a razão e a emoção, procurando multiplicar as perguntas e se distanciar dos limites confortáveis, com sensibilidade para a escuta dos usuários e incorporando novas tecnologias de cuidado para uma prática de enfermagem humanizada por meio de estratégias diferenciadas e inovadoras^(3)^.

Para efetivar o cuidado em saúde mental nessa perspectiva, a prática clínica precisa estar embasada em referenciais teóricos que reforcem o modelo de atenção psicossocial centrada no indivíduo e suas complexidades, contrapondo-se ao modelo tradicional de atenção psiquiátrica, pautado por ações restritas à medicalização, diagnóstico, sinais e sintomas.

As teorias de enfermagem, especificamente, orientam a prática de cuidado dos enfermeiros em suas diferentes funções, dando visibilidade aos fundamentos filosóficos de enfermagem, e são ferramentas importantes de suporte a essa mudança de paradigma.

Para fundamentar e qualificar o cuidado clínico de enfermagem em saúde mental, surgiu, em meados dos anos 1990, o *Tidal Model* (Modelo das Marés), criado pelo enfermeiro psiquiátrico escocês Phil Barker, que defende a existência de um trabalho cooperativo entre enfermeiro e paciente, no qual o profissional assume o papel de aprendiz sobre a experiência vivida pela pessoa.

O *Tidal Model* enfatiza a necessidade de descobrir o que a saúde mental significa para cada sujeito, já que seu significado varia de uma pessoa para outra, de modo único. Considera que tudo está marcado na história de vida das pessoas, a qual apresenta todas as mudanças que ocorreram durante a viagem desde o nascimento, e como responderam a cada uma delas. Para o *Tidal Model*, o resgate dessas histórias pessoais e dos problemas como seres humanos representa o primeiro passo para a recuperação do controle sobre a vida^(4)^. Trata-se de uma teoria que incita mudanças teórico-conceituais, desenvolvida especificamente para a prática de enfermagem em saúde mental, que permite aos enfermeiros qualificar o cuidado às pessoas em sofrimento e, consequentemente, fortalecer a Política de Atenção Psicossocial no país^(5)^. Afasta-se do paradigma convencional, focado na figura do especialista, com abordagem farmacológica redutiva, situando a pessoa no centro dos cuidados de enfermagem e permitindo que ela se sinta partícipe em todo o processo de recuperação de sua saúde mental.

Além disso, propõe trabalhar e gerenciar “a pessoa com risco”, e não o “risco” propriamente dito, reorientando as práticas de enfermagem para um caminho de integralidade e, consequentemente, com respostas terapêuticas. O *Tidal Model*, com sua abordagem holística e centrada na pessoa, representa uma evolução significativa em relação às teorias clássicas de enfermagem, como as de Peplau e Travelbee. Embora compartilhe com essas teorias o foco na relação terapêutica e na experiência do paciente, o *Tidal Model* apresenta características únicas que o diferenciam e o tornam uma ferramenta valiosa para a prática contemporânea de enfermagem em saúde mental. Enquanto as teorias de Peplau e Travelbee forneceram bases sólidas para a compreensão da relação enfermeiro-paciente e do significado da doença, o *Tidal Model* oferece uma perspectiva mais ampla e complexa, que leva em consideração o contexto social, cultural e histórico do paciente, enfatizando a importância da narrativa, da mudança e do protagonismo da pessoa.

O *Tidal Model* não substitui as teorias clássicas, mas as complementa, em uma perspectiva mais abrangente e atualizada, oferecendo ferramentas práticas para avaliação e intervenção em enfermagem, permitindo aos enfermeiros oferecer um cuidado mais humanizado, eficaz e centrado nas necessidades da pessoa. Por esse caráter diferenciado, e considerando a multidimensionalidade do ser em sofrimento psíquico, percebem-se, nos pressupostos do *Tidal Model*, elementos de aproximação com o pensamento complexo proposto por Edgar Morin, que afirma a necessidade de uma nova compreensão de sujeito que reconheça, potencialmente, que toda pessoa é sujeito, ator e autor capaz de cognição, escolha e decisão, em um meio social de relações intersubjetivas^(6)^.

A complexidade permite a elaboração conjunta de elementos distintos ou diferentes, em direção a uma compreensão global dos fenômenos, propiciando o entendimento da saúde enquanto fenômeno complexo nas instâncias individual, social e estrutural^(7)^. A perspectiva teórica da complexidade auxilia a repensar a ontologia do ser humano, do enfermeiro e do cuidado, e a reformular o conhecimento de enfermagem, superando o paradigma da simplificação e fragmentação, em busca de novos modos de cuidar, ensinar e gerir o cuidado^(8)^.

Diante disso, surgiu o interesse em refletir sobre a articulação entre o *Tidal Model* e a Teoria da Complexidade, evidenciando a adequação do *Tidal Model* para o cuidado clínico de enfermagem em saúde mental segundo as diretrizes do modelo de atenção psicossocial. Com isso, espera-se auxiliar os enfermeiros quanto ao uso de referencial próprio da disciplina de enfermagem na assistência em saúde mental, fortalecendo sua identidade profissional. Vai ao encontro da recomendação do Conselho Federal de Enfermagem (COFEN), que, por meio da Resolução COFEN n° 678, de 19 de agosto de 2021^(9)^, estabelece as ações do enfermeiro generalista no contexto da saúde mental e preconiza a utilização de modelos teóricos para fundamentar e sistematizar ações de cuidado em saúde mental. Ademais, colabora na busca de um referencial que apresente uma nova concepção sobre a saúde mental e sofrimento psíquico consonante com os pressupostos da desinstitucionalização.

## MÉTODO

Trata-se de reflexão teórica, de abordagem qualitativa, que surgiu a partir de discussões realizadas na disciplina análise crítica dos cuidados clínicos em enfermagem e saúde, oferecida no curso de doutorado do programa de pós-graduação cuidados clínicos em enfermagem e saúde da Universidade Estadual do Ceará. Foram utilizados os conceitos-chave da Teoria da Complexidade, de Edgar Morin, imbricados no *Tidal Model*, de Phil Barker, ressaltando os benefícios da aplicação do modelo na prática do cuidado clínico de enfermagem em saúde mental, evidenciando a consonância do *Tidal Model* com as diretrizes do modelo de atenção psicossocial preconizadas pelo Ministério da Saúde.

A proposta de articular a Teoria da Complexidade com o *Tidal Model* oferece uma perspectiva rica para compreender os desafios e as oportunidades enfrentadas em um mundo cada vez mais interconectado e complexo. Ambas as teorias, embora oriundas de campos distintos, compartilham a premissa de que os sistemas, sejam eles sociais, organizacionais ou individuais, são intrinsecamente complexos e não podem ser reduzidos a uma simples soma de suas partes.

Existem diversos outros pontos de convergência e sinergia entre as duas teorias, além da ênfase em uma visão sistêmica, com destaque para a dinâmica e a mudança como características inerentes aos sistemas e a necessidade de desenvolver abordagens mais eficazes para lidar com a incerteza e a complexidade, em um processo de aprendizagem contínua, que vai ao encontro das premissas do cuidado integral no campo da saúde mental.

A leitura e o estudo dos livros “Introdução ao pensamento complexo”^(10)^, de Edgar Morin, e *“El Modelo Tidal: salud mental, reinvidicación y recuperación”* ^(4)^, de Phil Barker e Poppy Buchanan-Barker, nos quais os autores descrevem suas respectivas teorias, acrescidas da leitura de outras produções textuais correlatas, permitiram estruturar esta reflexão teórica, organizada em três eixos: “O *Tidal Model*”, que apresenta brevemente os pressupostos do *Tidal Model*, possibilitando uma aproximação geral com o pensamento de Phil Barker; “Considerações sobre o pensamento complexo de Edgar Morin”, que por sua vez também busca esclarecer conceitos-chave da Teoria da Complexidade; e “O *Tidal Model* sob o olhar da complexidade: implicações ao cuidado clínico de enfermagem em saúde mental”, que demonstra a articulação entre os dois modelos, identificando a inter-relação entre o *Tidal Model* e as diretrizes do modelo de atenção psicossocial, apresentando o uso da teoria como fomento para o cuidado clínico de enfermagem em saúde mental sob novas perspectivas.

## O *TIDAL MODEL*

O *Tidal Model* é uma teoria de enfermagem que propõe o cuidado centrado na pessoa, auxiliando-a na descoberta dos problemas da vida diária que são a causa de seu sofrimento psíquico, bem como na mobilização de recursos próprios para dar respostas a eles, recuperando sua saúde mental.

A teoria é reconhecida internacionalmente e tem ampla aplicação em contextos diversos, como ambulatórios, clínicas de reabilitação, atenção domiciliar, entre outros. Além disso, permite um trabalho individual e coletivo direcionado a vários públicos, como crianças, adolescentes, idosos e famílias.

Estudo publicado em 2022 sobre a aplicabilidade do *Tidal Model* por enfermeiros em serviços de saúde mental identifica como resultados da aplicação/incorporação dessa teoria nos cuidados de enfermagem: a redução da agressão física, da violência dirigida a outros ou a si mesmo e o assédio; a redução do uso de substâncias psicoativas; o aumento da resiliência de mulheres vítimas de violência; a melhoria nas estratégias de *coping* e autoestima da pessoa; a satisfação do enfermeiro; a avaliação positiva do usuário do serviço acerca da assistência prestada, a amplitude da avaliação do enfermeiro relacionada ao quadro de saúde da pessoa com ideação suicida; a redução do tempo de internação e do uso de contenção; a aproximação do enfermeiro à pessoa que precisa de cuidados; e a valorização da prática de enfermagem em saúde mental^(5)^.

O modelo de Phil Barker enriquece o corpo teórico de enfermagem, na medida em que redefine o paradigma do cuidado em saúde mental, oportunizando o trabalho interprofissional e garantindo autonomia ao enfermeiro e aos sujeitos em sofrimento psíquico no processo de cuidado^(11)^. Utilizando a metáfora das águas, a teoria postula que a experiência humana tem natureza fluida, e considera a vida como um oceano de experiências, na qual a mudança é um processo constante e ilimitado. Nessa viagem em evolução, de exploração e descobrimento, depara-se com problemas imprevisíveis, com os quais nem sempre consegue lidar. Nesse ponto, vivencia-se um naufrágio emocional, em que surgem manifestações públicas de que algo não vai bem, embora a experiência de sofrimento siga invisível no domínio do “Eu” (*Self*). Para se aproximar do significado dessa experiência pessoal, ao enfermeiro cabe a missão de estabelecer uma comunicação sincera e autêntica, com a valorização da expressão da pessoa, sua voz, sua linguagem, de maneira que a preocupação humana seja seu guia, em um sentimento de conexão (relação ponte) desencadeado por meio da ferramenta-chave do processo terapêutico: o relacionamento interpessoal.

O *Tidal Model* apresenta seis princípios orientadores: a virtude da curiosidade, que consiste em investigar o que motivou a busca por cuidados em saúde mental; o poder da desenvoltura, que busca desvelar de que maneira a pessoa convive ou coexiste com o sofrimento/adoecimento; o valor do respeito aos desejos da pessoa em um processo de cuidado pautado na colaboração ativa, centrado na pessoa; o paradoxo, crise como oportunidade que enxerga a crise como oportunidade para mudança; a sabedoria cotidiana, que estabelece metas em que são traçados pequenos passos diários para o alcance de objetivos específicos; e a busca da elegância, na elaboração de um plano de cuidados de enfermagem que contenha ações simples para que a pessoa experimente uma mudança com foco naquilo que é necessário^(4)^.

O modelo está embasado em quatro perguntas fundamentais: por que isso, por que agora? Atentar ao que a pessoa está experimentando no momento. O que funciona? O que funcionou ou pode funcionar para solucionar o problema sob a perspectiva da pessoa? Qual é a “teoria pessoal” da pessoa? Como ela compreende e explica o que está acontecendo e como limitar as restrições? Fazer o mínimo possível pela pessoa, permitir que ela mesmo faça^(4)^.

O *Tidal Model* elenca dez compromissos que são a essência da sua aplicação na prática profissional: valorizar a voz dos sujeitos, permitindo que expressem sua experiência e contem sua história; respeitar sua língua, encorajando-os a falar com suas próprias palavras como compreendem o que estão experimentando; tornar-se aprendiz, aprendendo com o outro; utilizar o “*kit* de ferramentas” disponíveis, ajudando a pessoa a identificar aquilo “que funcionou” ou “o que pode funcionar” para ajudá-la a lidar com os problemas que vivencia; desenvolver a capacidade de dar um passo além, detectando o que precisa ser feito “agora” para ajudar a prosseguir; dar o presente do tempo; desenvolver a curiosidade genuína, identificando as informações necessárias; saber que a mudança é constante; revelar sabedoria pessoal, reconhecendo as potencialidades da pessoa em seu processo de recuperação; e ser transparente^(11)^. Atender a cada um desses compromissos exige do enfermeiro competências específicas que precisam ser desenvolvidas para gerar uma situação de aprendizagem com o outro e de corresponsabilização pelo cuidado.

Por fim, o cuidado *continuum* proposto pelo *Tidal Model* orienta que tipo de cuidado a pessoa em sofrimento psíquico precisa receber, conforme o seu estado e necessidades de saúde, desde cuidados imediatos, diante de uma tempestade ou afogamento, para que a pessoa possa restabelecer o seu percurso, perpassando os cuidados de transição, que auxiliam a pessoa a continuar sua jornada, compreendendo o que aconteceu para que ela saísse do seu percurso, até cuidados para o desenvolvimento, que habilitam a pessoa a lidar com essas situações no futuro, até que consiga agir de forma independente do serviço de saúde, retomando suas atividades cotidianas^(4)^.

## CONSIDERAÇÕES SOBRE O PENSAMENTO COMPLEXO DE EDGAR MORIN

A palavra “complexidade” origina-se do termo grego *complexus*, traduzível como “o que tece junto”. Entretanto, no senso comum, passou a ser empregada como sinônimo de “complicado”, “difícil de entender”.

No denominado pensamento complexo do filósofo francês Edgar Morin, que vem sendo desenvolvido e ampliado desde a década de 1960, o qual vê o mundo como um todo indissociável, a complexidade é entendida como “o tecido de acontecimentos, ações, interações, retroações, determinações, acasos que constituem o mundo fenomênico, e se apresenta com os traços inquietantes do emaranhado, do inextricável, da desordem, da ambiguidade, da incerteza”^(10)^.

Edgar Morin utiliza princípios da Teoria da Informação, Teoria da Cibernética, Teoria dos Sistemas, Teoria da Auto-Organização e Teoria da Microfísica, bem como o conceito de sistema aberto, que ressaltam a relação sujeito/objeto, a qual colabora para a mudança paradigmática que propõe agregação dos saberes em detrimento da fragmentação e do reducionismo da ciência clássica^(12)^.

Em seu modelo, afirma que a incerteza e as contradições fazem parte da vida e da condição humana, e que a religação dos seres e dos saberes só é possível por meio da solidariedade e da ética.

A Teoria da Complexidade de Morin deseja exercer um pensamento capaz de lidar, dialogar e negociar com o real, priorizando as articulações entre as disciplinas desmembradas pelo pensamento disjuntivo, que isola o que separa e oculta o que religa. Para tanto, propõe substituir o modo de pensar dicotômico das dualidades sujeito-objeto, parte-todo, razão-emoção, entre outros, da visão cartesiana por um modo de pensar articulado, abandonando o paradigma de um universo mecanicista, entendendo o mundo na perspectiva dos sistemas complexos.

São conceitos-chave da teoria: dialógica, que compreende os fenômenos como antagônicos, concorrentes e complementares de forma simultânea; recursividade organizacional, que postula que os efeitos de um processo são também seus coprodutores (auto-organização e autoprodução); holograma, que afirma que a parte está no todo, como o todo está na parte e tetragrama da complexidade, representado pelo paradigma ordem-desordem- interação-organização^(10)^. Nessa perspectiva, é possível manter a dualidade presente na unidade e conceber a ordem e a desordem como conceitos relativos e relacionais, ambas cooperando para organização do universo.

A Teoria da Complexidade foi difundida em todo o mundo, e seus princípios são aplicados amplamente em estudos nos campos da educação, saúde e organizações, entre outros, abrindo novas perspectivas para o conhecimento científico em geral e favorecendo a criação de abordagens inovadoras para a solução de problemas.

## O TIDAL MODEL SOB O OLHAR DA COMPLEXIDADE: IMPLICAÇÕES PARA O CUIDADO CLÍNICO DE ENFERMAGEM EM SAÚDE MENTAL

Integrar os preceitos da Teoria da Complexidade, tal como apresentados por Edgar Morin, com o *Tidal Model* de Phill Barker, relacionando-os às diretrizes do modelo de atenção psicossocial, é de suma importância, pois promove um aprofundamento conceitual que vai além da simples comparação entre teorias, enriquecendo o conhecimento na área da enfermagem e ampliando a compreensão dos fenômenos complexos que permeiam o cuidado em saúde mental.

A RAPS, que aspira à promoção da cidadania e da inclusão social da pessoa em adoecimento mental, bem como à integralidade e à continuidade de seu cuidado, é exemplo vivo de uma reforma paradigmática e do pensamento, corroborando a perspectiva de Morin de que a hiperespecialização, que fragmenta o global e dilui o essencial, deve dar lugar a um pensamento complexo, que seja capaz de perceber o processo interacional entre as partes e o todo e, assim, solucionar problemas multidimensionais.

Ao adotar uma perspectiva complexa, os profissionais de enfermagem são encorajados a transcender modelos reducionistas e fragmentados, reconhecendo a interdependência entre os diversos fatores que influenciam o adoecimento mental. Essa abordagem permite uma prática mais integral e humanizada, alinhada com os princípios da RAPS.

A RAPS estabelece-se em múltiplas conexões e inter- relações no território. Sob o olhar da complexidade, é possível distinguir a objetividade das leis e portarias ministeriais que a regulamentam e a subjetividade de seus atores e a interação nos serviços em toda sua dinamicidade. Nesse emaranhado vivo, pode-se perceber a ordem e desordem em interação constante, o que possibilita que a rede se concretize, auto-organize-se e avance^(12)^. O *Tidal Model*, por sua vez, quando analisado sob a ótica da Teoria da Complexidade, oferece uma estrutura teórica robusta para a prática de enfermagem em saúde mental. Considerando que a enfermidade mental engloba uma grande variedade de problemas da vida humana, o *Tidal Model* enfatiza que o enfermeiro deve mobilizar a essência do cuidado em enfermagem, que é fornecer apoio à pessoa, e afirma que a enfermagem autêntica tem lugar quando a pessoa começa sua experiência de crescimento e desenvolvimento.

As enfermeiras devem ser flexíveis, pragmáticas e, sobretudo, criativas no cuidado em saúde mental, sendo que elas mesmas são as principais ferramentas terapêuticas, auxiliando a pessoa a contextualizar seu processo de saúde-doença e identificar o que funciona para ela, desenvolvendo a sua teoria pessoal sobre o que sente, facilitando, sem grandes interferências, a recuperação de sua saúde mental, em um movimento de constante valorização humana^(13)^. Assim, a ética da compreensão humana começa quando se concebe e sente os seres humanos como sujeitos, o que torna os profissionais sensíveis a suas alegrias e sofrimentos, representando um requisito fundamental dos tempos atuais de incompreensão generalizada^(14)^.

Percebendo essas aproximações entre as duas teorias, propõe-se aqui uma discussão sobre os princípios da complexidade aplicados ao *Tidal Model* e sua adequação às diretrizes da RAPS, o que se expõe, ilustrativamente, no Quadro 1, para melhor visualização.

**Quadro 1:** Aplicação dos princípios da complexidade no *Tidal Model* e nas diretrizes da Rede de Atenção Psicossocial – Fortaleza, CE, Brasil, 2025.

Princípio da complexidade	Aplicação no *Tidal Model*	Diretrizes da Rede de Atenção Psicossocial(1)
**Dialógica**	– Admitem-se antagonismos complementares: saúde x doença mental; enfermeiro x pessoa em sofrimento psíquico; todo x partes (domínios do *Self*, do Mundo e dos Outros); – Em seu quarto princípio orientador, visualiza a crise como oportunidade para mudança.	– “O respeito aos direitos humanos, garantindo a autonomia e a liberdade das pessoas”; – “A atenção humanizada e centrada nas necessidades das pessoas”; – “A promoção da equidade, reconhecendo os determinantes sociais da saúde”.
**Recursividade organizacional**	– A mudança é um processo constante; – Conhecimento e descobertas sobre a relação da pessoa com a saúde e a enfermidade, construídos e desvelados em colaboração com o enfermeiro, auxiliam a formar novos conhecimentos, os quais geram novas perspectivas a respeito do tema.	– “O respeito aos direitos humanos, garantindo a autonomia e a liberdade das pessoas”; – “A atenção humanizada e centrada nas necessidades das pessoas”; – “Diversificação das estratégias de cuidado”.
**Holograma**	– A pessoa é representada e deve ser compreendida em três domínios pessoais: o *Self*, o Mundo e os Outros.	– “A promoção da equidade, reconhecendo os determinantes sociais da saúde”;
**Tetragrama da complexidade**	– Considera que a imprevisibilidade da natureza é similar à vida humana, comparando-a ao fluxo da água, ao movimento de ir e vir das marés, extraindo sua metáfora filosófica central da Teoria do Caos. – A vida e sua imprevisibilidade são ameaçadoras, e os problemas humanos do cotidiano advêm de interações complexas da pessoa com eles, possibilitando a instalação do caos.	– “O combate a estigmas e preconceitos; a garantia de acesso e da qualidade dos serviços, ofertando cuidado integral e assistência multiprofissional, sob a lógica interdisciplinar”.

Fonte: elaboração das autoras.

A dialógica permeia o *Tidal Model* na medida em que conceitos essencialmente antagônicos por definição são expostos em complementariedade. Como em uma conexão contínua entre contrários, como saúde e doença mental, enfermeiro e pessoa em sofrimento psíquico, o todo (representado pela pessoa) e as partes (os domínios do *Self*, do Mundo e dos Outros), aparecem interligados, caracterizando a enfermagem como uma atividade interativa relacionada ao desenvolvimento e crescimento humano.

Além disso, o princípio orientador do *Tidal Model*, que destaca a crise como oportunidade de mudança, coloca o enfermeiro frente a frente com a dialógica ressaltada por Morin, na qual “o ser, organizador-de-si, para organizar-se, necessita fechar-se em relação ao ecossistema, mas também estar aberto em relação a ele, pois é nesse processo interativo que ele se organiza, expressando a relação indissociável entre sujeito e mundo, isto é, o mundo está no sujeito e o sujeito no mundo” ^(14)^. Portanto, permeado pela complexidade, o *Tidal Model* reafirma o relacionamento interpessoal como ferramenta imprescindível ao sucesso do cuidado de enfermagem em saúde mental. Vai ao encontro do paradigma do cuidado psicossocial, que recomenda assistir a pessoa em sofrimento psíquico prioritariamente em serviços de base territorial e compreender seu modo de vida e suas relações, permitindo que mantenha sua autonomia e liberdade.

A recursividade organizacional está presente na principal premissa do *Tidal Model*, segundo a qual a mudança é constante, e ao enfermeiro cabe identificar que tipo de mudança representa um passo adiante no processo de recuperação da saúde mental, desenvolvendo na pessoa a consciência de que mesmo os pequenos passos são capazes de promover transformações. Nesse ponto, há uma consonância com o pressuposto de Edgar Morin, segundo o qual todos os resultados de um processo também são seus coprodutores, em um circuito auto-constitutivo e auto-organizador que estimula ações dinâmicas, interativas e recursivas, abandonando a concepção linear de causa e efeito.

A interlocução das teorias reforça a ideia de que o percurso para a recuperação da saúde mental é singular. Desse modo, cada sujeito encontrará o melhor caminho no fluxo da vida para alcançar seu bem-estar, o que vai ao encontro da atenção humanizada e centrada nas necessidades das pessoas, propondo estratégias diversificadas de cuidado rumo à integralidade.

No *Tidal Model*, está expressa ainda a necessidade de compreender a pessoa na totalidade, em três domínios: o *Self*, que é o lugar privado de experiências humanas, conhecido completamente apenas pela pessoa; o Mundo, no qual a pessoa divide algumas vivências do domínio do *Self* com os outros no mundo social; e os Outros, que é o cenário no qual a pessoa compartilha a vida diária com a família, os amigos, os vizinhos, os colegas de trabalho, os profissionais, *etc*., afetando-os e sendo afetada por eles^(13)^.

Essa premissa corrobora o princípio hologramático, que ultrapassa o reducionismo, que só visualiza as partes, e o holismo, que só enxerga o todo, admitindo que a parte está no todo, mas também que o todo está na parte. Reconhece que é possível aumentar o entendimento das partes pelo todo e do todo pelas partes, deslocando-se de um pensamento disjuntivo e redutor para o pensamento complexo. Contextualizar, portanto, permite atribuir sentido ao cuidado humano, fazendo florescer as habilidades necessárias para o cuidado do outro e da vida, muito além das competências cognitivas, pela compreensão de espaços onde tudo é construído e reconstruído todo o tempo, em um processo permeado por variadas emoções^(15)^. Assim, a compreensão humana propulsiona a harmonia entre pessoas distintas, seja por suas escolhas, origem étnica, orientação sexual ou experiência de sofrimento, auxiliando a trilhar o caminho para equidade, considerando os determinantes sociais da saúde, conforme proposto pelo modelo de atenção psicossocial.

Por fim, o tetragrama da complexidade perpassa o *Tidal Model* ao afirmar que somente com o auxílio do paradoxo ordem-desordem é possível promover a organização. O conceito de ordem ultrapassa a ideia determinista de estabilidade, apoiando-se na ideia das interações mútuas, considerando que nada existe sem influências internas e externas e sua interdependência. Além disso, a desordem abandona a ideia do acaso, apesar de sempre admiti-lo, pois, para haver desordem, é preciso haver “separação, instabilidades e inconstâncias”^(14)^. Portanto, a Teoria da Complexidade toma como base um pensamento que considera todas as influências, internas e externas, destacando a incerteza como princípio norteador, e não propõe sua eliminação; ao contrário, sugere ser necessário compreender a contradição e o imprevisível a partir da coexistência entre eles^(14)^.

A integração dessas teorias favorece: a prática profissional do enfermeiro, que implica personalização do cuidado, reconhecendo a singularidade de cada pessoa, sua história de vida e contexto social; a promoção da autonomia, encorajando a participação do paciente em seu processo de recuperação, valorizando sua voz e experiência; o uso de flexibilidade e criatividade para navegar em situações de incerteza e adaptar suas abordagens; o fortalecimento do relacionamento terapêutico, em que a interação enfermeiro-paciente se torna central, promovendo um vínculo baseado na compreensão e respeito mútuos.

Assim, o pensamento complexo e o *Tidal Model* facilitam elaborar ações de cuidado em ambientes incertos como em face de crises, pois essas ações são produzidas pela análise concomitante de condições definidas (ordem) e indefinidas (desordem), e é dessa avaliação que surge um plano de cuidados em saúde mental complexo que respeita as histórias de vida e os significados de saúde e doença para as pessoas em sofrimento psíquico.

A concepção linear, determinista e apoiada na certeza precisa ser reformulada para criar modelos que contemplem a complexidade da vida. Isso implica adotar princípios teóricos em sintonia com os avanços da enfermagem como ciência, arte e profissão. Nesse sentido, a complexidade representa uma abordagem que ressalta a inter e a transdisciplinaridade como alternativas para reestruturar o conhecimento na contemporaneidade^(8)^.

A abordagem da subjetividade precisa aproximar a enfermagem de competências e habilidades essenciais para a humanização do cuidado, visto que o trabalho clínico e humanizado na saúde mental exige tempo para acolhimento, escuta qualificada, atenção e relacionamento interpessoal com os sujeitos que sofrem, tendo em vista a reorientação do cuidado na perspectiva de privilegiar as possibilidades e potencialidades dos sujeitos com vistas à reintegração social^(16)^.

A Teoria da Complexidade é utilizada, em especial, para compreender as relações e fenômenos envolvidos no cuidado de enfermagem em sua totalidade e em diferentes fases do desenvolvimento humano, enfatizando a necessidade de incorporar múltiplos olhares para sua execução^(17)^. Considerando a prática de enfermagem amplamente relacionada à complexidade do Ser, o enfermeiro, como um ser social detentor de conhecimentos técnicos científicos, deve refletir sobre novas formas de cuidado que possam abranger a dinamicidade e integralidade, articulando redes de cuidado e interconexões que dependem de sua atuação^(17)^.

A articulação entre a Teoria da Complexidade e o *Tidal Model*: permite compreender os fenômenos em sua complexidade, considerando as interconexões entre os diferentes elementos (visão sistêmica); facilita a adaptação a cenários em constante mudança e criação de soluções mais flexíveis (adaptabilidade); promove a colaboração entre diferentes atores e construção de soluções conjuntas (trabalho colaborativo); e contribui para a construção de sistemas e organizações mais resilientes e capazes de lidar com a incerteza (resiliência).

Como aplicação prática ao cuidado clínico de enfermagem em saúde mental, tem-se: o estabelecimento de relacionamentos interpessoais mais significativos, compreendendo a multiplicidade de fatores envolvidos na experiência de sofrimento dos sujeitos, capazes de propiciar maior vinculação dos usuários e familiares aos serviços e, consequentemente, maior reconhecimento e satisfação profissional dos enfermeiros; a possibilidade de elaboração de Projetos Terapêuticos Singulares mais específicos e eficazes, considerando a centralidade na pessoa e em sua história; e a criação de novas estratégias individuais e coletivas para o trabalho multidisciplinar de maneira integrada, com um olhar ampliado, tão importante para dar conta das múltiplas demandas apresentadas pelas pessoas em sofrimento psíquico.

A interlocução entre a Teoria da Complexidade e o *Tidal Model* permite aos enfermeiros desenvolver ações que colaborem para as premissas de lutar contra estigmas e preconceitos, assegurar o acesso e a qualidade dos serviços, e oferecer cuidado integral e assistência multiprofissional sob o olhar interdisciplinar, fortalecendo o modelo de atenção psicossocial.

## CONCLUSÃO

Na intenção de consolidar a enfermagem em saúde mental, prática holística, inclusiva, positiva e colaborativa, em detrimento da enfermagem psiquiátrica, paternalista, coercitiva, negativa e orientada para a doença, urge oferecer um cuidado clínico em saúde mental que supere a visão biologicista, dicotômica e fragmentada, pautada pelo uso de medicamentos, a fim de compreender a pessoa em sofrimento psíquico de maneira multidimensional, conectando o sujeito a sua dimensão sociocultural. Para favorecer essa visão ampliada, foram encontrados, na proposta do *Tidal Model* de Phill Barker, elementos que dialogam com a Teoria da Complexidade de Edgar Morin, que vão ao encontro das diretrizes do modelo de atenção psicossocial, apresentando-se, portanto, como teoria capaz de embasar e direcionar a prática de saúde mental e suas complexidades.

Sugere-se aos enfermeiros aprofundar o estudo de teorias de enfermagem voltadas para o cuidado em saúde mental, refletindo criticamente sobre elas, contribuindo para aperfeiçoar modelos, bem como adaptar e ampliar sua aplicação em diferentes contextos como forma de favorecer seu protagonismo como agente terapêutico, colaborando para o protagonismo dos sujeitos em sofrimento psíquico e alcançando a consolidação da tão almejada atenção psicossocial no sistema de saúde.

O *Tidal Model* e a Teoria da Complexidade, embora distintos, convergem em seus princípios fundamentais para o cuidado em saúde mental. Ao integrar esses conceitos, cria-se uma estrutura teórica mais robusta que permite compreender o ser humano em sua totalidade, reconhecendo a complexidade inerente aos processos de adoecimento e recuperação em saúde mental. Essa reflexão é crucial para superar modelos reducionistas e fragmentados, promovendo uma prática de enfermagem que valoriza a subjetividade, a inter-relação entre indivíduo e ambiente, e a constante transformação presente na vida humana.

# Translation: *Tidals Model* a la luz de la complejidad: aportes a la atención clínica de enfermería en salud mental

**DOI:** 10.1590/1980-220X-REEUSP-2024-0377es

**Authors:** Natália Rocha Chagas Comaru, Ana Ruth Macêdo Monteiro, Lucia de Fatima da Silva, Maria Célia de Freitas, Maria Vilani Cavalcante Guedes

**Affiliations:** 1 Universidade Estadual do Ceará, Fortaleza, CE, Brasil.

**Keywords:** Enfermería, Salud Mental, Atención a la Salud Mental, Filosofía en Enfermería, Teoría de Enfermería

## Abstract

Este es un ensayo teórico que tiene como objetivo reflexionar sobre las articulaciones entre el Modelo Tidals de Phil Barker y la Teoría de la Complejidad de Edgar Morin, destacando la idoneidad del Modelo Tidals para la atención clínica de enfermería en salud mental según las directrices del modelo de atención psicosocial. Se utilizaron los conceptos claves de la Teoría de la Complejidad (dialógica, recursividad organizacional, holograma y tetragrama) en conjunto con el Modelo Tidals, identificándose que este marco está alineado con los siguientes lineamientos de la atención psicosocial: respeto a los derechos humanos, atención humanizada, promoción de la equidad, diversificación de las estrategias de atención, combate al estigma y prejuicio, garantizar el acceso a los servicios y ofrecer atención integral y asistencia multidisciplinaria, bajo la lógica interdisciplinaria. El Modelo Tidals representa una alternativa a los modelos de atención tradicionales, con una propuesta de atención alineada con las políticas de salud pública actuales. Se sugiere una mayor apropiación de este marco por parte de los enfermeros, ampliando su aplicación a los más diversos públicos y contextos de atención a la salud mental.

## INTRODUCCIÓN

La incorporación de las acciones de salud mental al Sistema Único de Salud (SUS) representa una estrategia gubernamental destinada a consolidar la Reforma Psiquiátrica Brasileña, iniciada en la década de 1970 y que resultó en el cierre gradual de las instituciones psiquiátricas en el país. La lucha anti-asilar permitió reorientar la práctica asistencial en redes de atención, promoviendo la descentralización de los cuidados y una mayor vinculación entre los usuarios y los servicios de salud, quedando la hospitalización restringida a los pacientes para los que los recursos extrahospitalarios se muestran ineficaces.

Para ello, la Orden Ministerial GM/MS 3088/2011^(1)^ creó la Red de Atención Psicosocial (RAPS), que ofrece atención integral, de forma articulada y en varios puntos de atención dentro del SUS, a las personas en situación de malestares psicológicos y con necesidades derivadas del uso nocivo de alcohol y otras drogas.

La RAPS tiene las siguientes directrices «el respeto a los derechos humanos, garantizando la autonomía y la libertad de las personas; la promoción de la equidad, reconociendo los determinantes sociales de la salud; el combate al estigma y al prejuicio; la garantía del acceso y de la calidad de los servicios, ofreciendo atención integral y asistencia multiprofesional, bajo una lógica interdisciplinaria; la atención humanizada y centrada en las necesidades de las personas; la diversificación de las estrategias de atención; el desarrollo de actividades en el territorio, favoreciendo la inclusión social con vistas a promover la autonomía y el ejercicio de la ciudadanía; el desarrollo de estrategias de Reducción de Daños; el énfasis en los servicios de base territorial y comunitaria, con la participación y el control social de los usuarios y sus familias; la organización de los servicios en una red regionalizada de atención a la salud, con el establecimiento de acciones intersectoriales para garantizar la atención integral; la promoción de estrategias de educación permanente; y el desarrollo de la lógica de atención a las personas con trastornos mentales y con necesidades derivadas del consumo de crack, alcohol y otras drogas, con la construcción del Proyecto Terapéutico Singular como eje central»^(1)^.

Como elementos estratégicos en la articulación del RAPS, destacamos los Centros de Atención Psicosocial, servicios regulados por la Ordenanza nº 336/2002^(2)^, que «ofrecen cuidados a la población, proporcionando seguimiento clínico y reinserción social de las personas con trastornos mentales a través del acceso al trabajo, al ocio, al ejercicio de los derechos civiles y al fortalecimiento de los lazos familiares y comunitarios».

Las enfermeras juegan un papel fundamental en estos centros, actuando como uno de los pilares del equipo multidisciplinar, proporcionando cuidados, escucha activa y educación en salud mental individual y colectiva, y participando en la gestión de los servicios mediante la elaboración de protocolos de atención y actividades en las unidades, así como promoviendo la coordinación entre usuarios, familias y otros servicios de la red sanitaria, garantizando la continuidad asistencial.

Este nuevo modelo de cuidados necesita ser absorbido por todos los profesionales, y las enfermeras deben estar preparadas para trabajar dentro de esta nueva lógica, abiertas a diversas posibilidades, dialogando con los diferentes discursos existentes sobre el sufrimiento psicológico, conviviendo con lo objetivo y lo subjetivo, con la razón y la emoción, buscando multiplicar las preguntas y distanciarse de los límites cómodos, con sensibilidad para escuchar a los usuarios e incorporando las nuevas tecnologías de cuidados para una práctica enfermera humanizada a través de estrategias diferenciadas e innovadoras^(3)^.

Para prestar cuidados de salud mental desde esta perspectiva, la práctica clínica necesita basarse en referencias teóricas que refuercen el modelo de atención psicosocial centrado en el individuo y sus complejidades, frente al modelo tradicional de atención psiquiátrica, basado en actuaciones restringidas a la medicalización, el diagnóstico, los signos y los síntomas.

Las teorías de enfermería, en particular, orientan la práctica asistencial de las enfermeras en sus diferentes roles, dando visibilidad a los fundamentos filosóficos de la enfermería, y son herramientas importantes para apoyar este cambio de paradigma.

Con el fin de apoyar y cualificar los cuidados clínicos de enfermería en salud mental, surgió a mediados de la década de 1990 el *Modelo Tidals (*Modelo de las Mareas*)*, creado por el enfermero psiquiátrico escocés Phil Barker, que defiende la existencia de un trabajo cooperativo entre la enfermera y el paciente, en el que el profesional asume el papel de aprendiz sobre la experiencia vivida por la persona. El *Modelo Tidals* hace hincapié en la necesidad de descubrir qué significa la salud mental para cada individuo, ya que su significado varía de una persona a otra de forma única. Considera que todo está marcado en la historia de vida de las personas, que presenta todos los cambios que se han producido durante el trayecto desde el nacimiento, y cómo han respondido a cada uno de ellos. Para El *Modelo Tidals*, recuperar estas historias y problemas personales como seres humanos es el primer paso para recuperar el control sobre la propia vida^(4)^. Es una teoría que incentiva cambios teórico-conceptuales, desarrollada específicamente para la práctica de enfermería en salud mental, que permite a las enfermeras cualificar el cuidado a las personas que sufren y, consecuentemente, fortalecer la Política de Atención Psicosocial del país^(5)^. Se aleja del paradigma convencional, centrado en la figura del especialista, con un enfoque farmacológico reductor, situando a la persona en el centro de los cuidados de enfermería y permitiendo que se sienta parte de todo el proceso de recuperación de su salud mental.

También propone trabajar y gestionar «la persona en riesgo» más que el «riesgo» en sí, reorientando las prácticas enfermeras hacia un camino de integralidad y, en consecuencia, las respuestas terapéuticas. El *Modelo Tidals*, con su enfoque holístico y centrado en la persona, representa una evolución significativa en relación con las teorías enfermeras clásicas, como las de Peplau y Travelbee. Aunque comparte con estas teorías un enfoque centrado en la relación terapéutica y en la experiencia del paciente, el *Modelo Tidals* tiene características únicas que lo diferencian y lo convierten en una herramienta valiosa para la práctica de la enfermería de salud mental contemporánea. Mientras que las teorías de Peplau y Travelbee proporcionaron bases sólidas para entender la relación enfermera-paciente y el significado de la enfermedad, El *Modelo* Tidals ofrece una perspectiva más amplia y compleja que tiene en cuenta el contexto social, cultural e histórico del paciente, enfatizando la importancia de la narrativa, el cambio y el protagonismo de la persona.

El *Modelo Tidals* no sustituye a las teorías clásicas, sino que las complementa en una perspectiva más completa y actualizada, ofreciendo herramientas prácticas para la valoración e intervención enfermera, permitiendo a las enfermeras ofrecer unos cuidados más humanizados, eficaces y centrados en las necesidades de la persona. Por este carácter distintivo, y considerando la multidimensionalidad de la persona en sufrimiento psicológico, podemos ver en los presupuestos dEl *Modelo Tidals* elementos más próximos al pensamiento complejo propuesto por Edgar Morin, que afirma la necesidad de una nueva comprensión del sujeto que reconozca potencialmente que toda persona es sujeto, actor y autor capaz de cognición, elección y decisión, en un ambiente social de relaciones intersubjetivas^(6)^.

La complejidad permite la elaboración conjunta de elementos distintos o diferentes, hacia una comprensión global de los fenómenos, proporcionando la comprensión de la salud como un fenómeno complejo en las esferas individual, social y estructural^(7)^. La perspectiva teórica de la complejidad ayuda a repensar la ontología del ser humano, de las enfermeras y del cuidado, y a reformular el conocimiento de enfermería, superando el paradigma de la simplificación y fragmentación, en busca de nuevas formas de cuidar, enseñar y gestionar el cuidado^(8)^. Dentro de este contexto, surgió el interés por investigar el tema.

Ante esto, hubo interés en reflexionar sobre la articulación entre El *Modelo Tidals* y la Teoría de la Complejidad, destacando la adecuación dEl *Modelo Tidals* para los cuidados clínicos de enfermería en salud mental según las directrices del modelo de atención psicosocial. Se espera que esto ayude a las enfermeras a utilizar su propia disciplina de enfermería en los cuidados de salud mental, fortaleciendo su identidad profesional. Está en consonancia con la recomendación del Consejo Federal de Enfermería (COFEN), que, a través de la Resolución COFEN n.º 678 del 19 de agosto de 2021^(9)^, establece las acciones de los enfermeros generalistas en el contexto de la salud mental y aboga por el uso de modelos teóricos para apoyar y sistematizar las acciones de atención en salud mental. Además, colabora en la búsqueda de un referente que presente una nueva concepción de la salud mental y del sufrimiento psíquico en consonancia con los presupuestos de la desinstitucionalización.

## MÉTODO

Se trata de una reflexión teórica, con abordaje cualitativo, que surgió de discusiones realizadas en la disciplina de análisis crítico del cuidado clínico en enfermería y salud, ofrecida en el curso de doctorado del programa de posgrado en cuidado clínico en enfermería y salud de la Universidad Estadual de Ceará. Se utilizaron los conceptos clave de la Teoría de la Complejidad de Edgar Morin, entrelazados con *el Modelo Tidals (Modelo de las Mareas)*de Phil Barker, destacando los beneficios de la aplicación del modelo a la práctica de los cuidados clínicos de enfermería en salud mental, mostrando que El *Modelo Tidals* está en consonancia con las directrices del modelo de atención psicosocial recomendado por el Ministerio de Salud.

La propuesta de articular la Teoría de la Complejidad con el Modelo *Tidals* ofrece una rica perspectiva para la comprensión de los desafíos y oportunidades enfrentados en un mundo cada vez más interconectado y complejo. Aunque ambas teorías proceden de campos diferentes, comparten la premisa de que los sistemas, ya sean sociales, organizativos o individuales, son intrínsecamente complejos y no pueden reducirse a una simple suma de sus partes.

Existen varios otros puntos de convergencia y sinergia entre ambas teorías, además del énfasis en una visión sistémica, destacando la dinámica y el cambio como características inherentes a los sistemas y la necesidad de desarrollar enfoques más eficaces para hacer frente a la incertidumbre y la complejidad, en un proceso de aprendizaje continuo, que responda a las premisas de la atención integral en el ámbito de la salud mental.

La lectura y estudio de los libros «Introducción al pensamiento complejo»^(10)^de Edgar Morin, y «El Modelo de las Mareas (Tidals): salud mental, re-invitación y recuperación»^(4)^de Phil Barker y Poppy Buchanan-Barker, en los que los autores describen sus respectivas teorías, además de la lectura de otros textos relacionados, permitieron estructurar esta reflexión teórica, organizada en tres ejes: «El *Modelo Tidals*“, que presenta brevemente los presupuestos dEl *Modelo Tidals*, permitiendo una aproximación general al pensamiento de Phil Barker; ”Consideraciones sobre el pensamiento complejo de Edgar Morin», que a su vez también pretende aclarar conceptos clave de la Teoría de la Complejidad; y «El *Modelo Tidals* desde la perspectiva de la complejidad»: Implicaciones para los cuidados clínicos de enfermería en salud mental», que demuestra la articulación entre los dos modelos, identificando la interrelación entre El *Modelo*Tidals y las directrices del modelo de atención psicosocial, presentando el uso de la teoría como potenciadora de los cuidados clínicos de enfermería en salud mental desde nuevas perspectivas.

## EL *MODELO TIDALS*

El *Modelo Tidals* es una teoría de enfermería que propone cuidados centrados en la persona, ayudando a las personas a descubrir los problemas de la vida cotidiana que son la causa de su malestar psicológico, así como a movilizar sus propios recursos para responder a ellos, recuperando así su salud mental.

La teoría es reconocida internacionalmente y tiene amplia aplicación en diversos contextos, como ambulatorios, clínicas de rehabilitación, atención domiciliaria, entre otros. También permite el trabajo individual y colectivo dirigido a diversos públicos, como niños, adolescentes, ancianos y familias. Un estudio publicado en 2022 sobre la aplicabilidad dEl *Modelo Tidals* por enfermeros en servicios de salud mental identifica los resultados de la aplicación/incorporación de esta teoría en los cuidados de enfermería reducción de las agresiones físicas, de la violencia dirigida a otros o a uno mismo y del acoso; reducción del consumo de sustancias psicoactivas; aumento de la resiliencia de las mujeres víctimas de violencia; mejora de las estrategias de *enfrentamiento* y de la autoestima de la persona; satisfacción de las enfermeras; la valoración positiva de los cuidados prestados por parte del usuario del servicio, la amplitud de la valoración de la enfermera en relación con el estado de salud de la persona con ideación suicida; la reducción del tiempo de hospitalización y del uso de sujeciones; la cercanía de la enfermera a la persona necesitada de cuidados; y la valorización de la práctica enfermera en salud mental^(5)^.

El modelo de Phil Barker enriquece el corpus de la teoría de enfermería en la medida en que redefine el paradigma de los cuidados en salud mental, ofreciendo oportunidades para el trabajo interprofesional y garantizando la autonomía de las enfermeras y de las personas con sufrimiento psíquico en el proceso de cuidados^(11)^. Utilizando la metáfora del agua, la teoría postula que la experiencia humana tiene una naturaleza fluida, y considera la vida como un océano de experiencias, en el que el cambio es un proceso constante e ilimitado. En este viaje evolutivo de exploración y descubrimiento, uno se topa con problemas imprevisibles que no siempre puede resolver. En este punto, experimentas un naufragio emocional, en el que hay manifestaciones públicas de que algo no va bien, aunque la experiencia de sufrimiento permanece invisible en el reino del «Yo» (*Self*). Para acercarse al significado de esta experiencia personal, la misión de la enfermera es establecer una comunicación sincera y auténtica, valorando la expresión de la persona, su voz, su lenguaje, para que la preocupación humana sea su guía, en un sentimiento de conexión (bridging relationship) desencadenado por la herramienta clave del proceso terapéutico: la relación interpersonal.

El *Modelo Tidals* tiene seis principios rectores: la virtud de la curiosidad, que consiste en indagar qué motivó la búsqueda de atención en salud mental; el poder de la recursividad, que busca descubrir cómo la persona vive o convive con el sufrimiento/enfermedad; el valor de respetar los deseos de la persona en un proceso de atención basado en la colaboración activa, centrada en la persona; la paradoja, la crisis como oportunidad, que ve en la crisis una oportunidad para el cambio; la sabiduría cotidiana, que establece metas en las que se dan pequeños pasos diarios para alcanzar objetivos concretos; y la búsqueda de la elegancia, en la elaboración de un plan de cuidados de enfermería que contenga acciones sencillas para que la persona experimente el cambio centrándose en lo necesario^(4)^.

El modelo se basa en cuatro preguntas fundamentales: ¿por qué esto, por qué ahora? Prestar atención a lo que la persona experimenta en ese momento. ¿Qué funciona? ¿Qué ha funcionado o podría funcionar para resolver el problema desde la perspectiva de la persona? ¿Cuál es la «teoría personal» de la persona? ¿Cómo entiende y explica lo que ocurre y cómo puede limitar las restricciones? Haga lo menos posible por la persona, permítale que lo haga por sí misma^(4)^.

El *Modelo Tidals* enumera diez compromisos que constituyen la esencia de su aplicación en la práctica profesional: valorar la voz de los sujetos, permitiéndoles expresar su experiencia y contar su historia; respetar su lenguaje, animándoles a decir con sus propias palabras cómo entienden lo que están experimentando; convertirse en un aprendiz, aprendiendo del otro; utilizar la «*caja de herramientas»*disponible, ayudando a la persona a identificar «lo que ha funcionado» o «lo que podría funcionar» para ayudarle a enfrentar los problemas que está experimentando; desarrollar la capacidad de ir un paso más allá, detectando lo que hay que hacer «ahora» para ayudarles a avanzar; regalar tiempo; desarrollar una curiosidad genuina, identificando la información necesaria; saber que el cambio es constante; revelar la sabiduría personal, reconociendo el potencial de la persona en su proceso de recuperación; y ser transparente^(11)^. Cumplir con cada uno de estos compromisos requiere de las enfermeras habilidades específicas que es necesario desarrollar para generar una situación de aprendizaje de los demás y de corresponsabilidad en el cuidado.

Por último, el *continuo de*cuidados propuesto por El *Modelo Tidals* orienta el tipo de atención que debe recibir la persona en situación de malestar psicológico, en función de su estado y necesidades de salud, desde los cuidados inmediatos, ante una tormenta o ahogamiento, para que la persona pueda restablecer su viaje, hasta los cuidados de transición, los cuidados de transición, que ayudan a la persona a continuar su camino, comprendiendo lo que sucedió para que abandonara su camino, hasta los cuidados de desarrollo, que capacitan a la persona para hacer frente a estas situaciones en el futuro, hasta que sea capaz de actuar de forma independiente del servicio sanitario, retomando sus actividades cotidianas^(4)^.

## CONSIDERACIONES SOBRE EL PENSAMIENTO COMPLEJO DE EDGAR MORIN

La palabra «complejidad» procede del término griego *complexus*, que se traduce como «lo que se entreteje». Sin embargo, en el sentido común, ha llegado a utilizarse como sinónimo de «complicado», «difícil de entender».

En el llamado pensamiento complejo del filósofo francés Edgar Morin, desarrollado y ampliado desde los años sesenta y que concibe el mundo como un todo inseparable, la complejidad se entiende como «el tejido de acontecimientos, acciones, interacciones, retroacciones, determinaciones, azares que constituyen el mundo fenoménico, y se presenta con los rasgos inquietantes de lo enredado, la inextricabilidad, el desorden, la ambigüedad, la incertidumbre»^(10)^.

Edgar Morin utiliza principios de la Teoría de la Información, la Teoría Cibernética, la Teoría de Sistemas, la Teoría de la Autoorganización y la Teoría de la Microfísica, así como el concepto de sistema abierto, que ponen de relieve la relación sujeto/objeto, lo que contribuye al cambio paradigmático que propone la agregación de conocimientos en detrimento de la fragmentación y el reduccionismo de la ciencia clásica^(12)^.

En su modelo, afirma que la incertidumbre y las contradicciones forman parte de la vida y de la condición humana, y que la reconexión de los seres y del conocimiento sólo es posible mediante la solidaridad y la ética.

La Teoría de la Complejidad de Morin quiere ejercer un pensamiento capaz de tratar, dialogar y negociar con la realidad, priorizando los vínculos entre disciplinas desmembradas por el pensamiento disyuntivo, que aísla lo que separa y oculta lo que conecta. Para ello, propone sustituir el pensamiento dicotómico de las dualidades sujeto-objeto, parte-todo, razón-emoción, entre otras, de la visión cartesiana por un pensamiento articulado, abandonando el paradigma de un universo mecanicista, entendiendo el mundo desde la perspectiva de los sistemas complejos.

Los conceptos clave de la teoría son: dialógico, que entiende los fenómenos como simultáneamente antagónicos, competidores y complementarios; recursividad organizativa, que postula que los efectos de un proceso son también sus coproductores (autoorganización y autoproducción); holograma, que afirma que la parte está en el todo, como el todo está en la parte; y el tetragrama de la complejidad, representado por el paradigma orden- desorden-interacción-organización^(10)^. Desde esta perspectiva, es posible mantener la dualidad presente en la unidad y concebir el orden y el desorden como conceptos relativos y relacionales, ambos cooperando para organizar el universo.

La Teoría de la Complejidad se ha extendido por todo el mundo y sus principios se aplican ampliamente en estudios en los campos de la educación, la salud y las organizaciones, entre otros, abriendo nuevas perspectivas para el conocimiento científico en general y favoreciendo la creación de enfoques innovadores para la resolución de problemas.

## EL *MODELO TIDALS* DESDE LA PERSPECTIVA DE LA COMPLEJIDAD: IMPLICACIONES PARA LOS CUIDADOS CLÍNICOS DE ENFERMERÍA EN SALUD MENTAL

Integrar los preceptos de la Teoría de la Complejidad, presentados por Edgar Morin, con El *Modelo* Tidals de Phill Barker, relacionándolos con las directrices del modelo de atención psicosocial, es de suma importancia, ya que promueve una profundización conceptual que va más allá de la simple comparación entre teorías, enriqueciendo el conocimiento en el campo de la enfermería y ampliando la comprensión de los fenómenos complejos que permean la atención en salud mental.

La RAPS, que aspira a promover la ciudadanía y la inclusión social de las personas con enfermedad mental, así como la integralidad y la continuidad de sus cuidados, es un ejemplo vivo de reforma paradigmática y de pensamiento, que corrobora la perspectiva de Morin de que la hiper especialización, que fragmenta lo global y diluye lo esencial, debe dar paso al pensamiento complejo, capaz de percibir el proceso interaccional entre las partes y el todo y resolver así problemas multidimensionales. Al adoptar una perspectiva compleja, se anima a los profesionales de enfermería a trascender los modelos reduccionistas y fragmentados, reconociendo la interdependencia entre los diversos factores que influyen en la enfermedad mental. Este enfoque permite una práctica más integral y humanizada, en consonancia con los principios de la RAPS.

La RAPS se basa en múltiples conexiones e interrelaciones en el territorio. Desde la perspectiva de la complejidad, es posible distinguir entre la objetividad de las leyes y ordenanzas ministeriales que lo regulan y la subjetividad de sus actores y la interacción en los servicios en todo su dinamismo. En esta maraña viva, se observa el orden y el desorden en constante interacción, lo que permite a la red materializarse, autoorganizarse y avanzar^(12)^. El *Modelo Tidals*, a su vez, cuando se analiza desde la perspectiva de la Teoría de la Complejidad, ofrece un marco teórico sólido para la práctica de la enfermería de salud mental. Teniendo en cuenta que la enfermedad mental abarca una amplia variedad de problemas de la vida humana, el Modelo *Tidals* hace hincapié en que las enfermeras deben movilizar la esencia de los cuidados de enfermería, que es proporcionar apoyo a la persona, y afirma que la auténtica enfermería tiene lugar cuando la persona comienza su experiencia de crecimiento y desarrollo.

La enfermera debe ser flexible, pragmática y, sobre todo, creativa en la atención a la salud mental, siendo ella misma la principal herramienta terapéutica, ayudando a la persona a contextualizar su proceso de salud-enfermedad e identificar lo que le funciona, desarrollando su teoría personal sobre lo que siente, facilitando, sin mayores interferencias, la recuperación de su salud mental, en un movimiento de constante valorización humana^(13)^. Así, la ética de la comprensión humana comienza cuando el ser humano es concebido y sentido como sujeto, lo que hace a los profesionales sensibles a sus alegrías y sufrimientos, representando un requisito fundamental en los tiempos actuales de incomprensión generalizada^(14)^. Teniendo en cuenta estas similitudes entre las dos teorías, proponemos una discusión de los principios de la complejidad aplicados al *Modelo Tidalss*y su adecuación a las directrices RAPS, que se ilustran en el Cuadro 1 para una mejor visualización.

**Cuadro 1:** Aplicación de los principios de complejidad en el *Modelo Tidals* y en las directrices de la Red de Atención Psicosocial – Fortaleza, CE, Brasil, 2025.

Principio de la complejidad	Aplicación en el *Modelo Tidals*	Directrices de la Red de Atención Psicosocial(1)
**Dialógica**	– Se aceptan antagonismos complementarios: salud frente a enfermedad mental; enfermera frente a persona con angustia psicológica; todo frente a partes (ámbitos del Yo, el Mundo y los Otros); – En su cuarto principio rector, visualiza la crisis como una oportunidad para el cambio.	– «Respeto de los derechos humanos, garantizando la autonomía y la libertad de las personas»; – «La atención humanizada centrada en las necesidades de las personas»; – «La promoción de la equidad, reconociendo los determinantes sociales de la salud».
**Recursividad organizacional**	– El cambio es un proceso constante; – Los conocimientos y descubrimientos sobre la relación de la persona con la salud y la enfermedad, construidos y desvelados en colaboración con la enfermera, ayudan a formar nuevos conocimientos, que generan nuevas perspectivas sobre el tema.	– «Respeto de los derechos humanos, garantizando la autonomía y la libertad de las personas»; – «Atención humanizada centrada en las necesidades de las personas»; – «Diversificación de las estrategias de atención».
**Holograma**	– La persona se representa y debe entenderse en tres ámbitos personales: el Yo, el Mundo y los Otros.	– «Promover la equidad, reconocer los determinantes sociales de la salud».
**Tetragrama de la complejidad**	– Considera que la imprevisibilidad de la naturaleza es similar a la vida humana, comparándola con el flujo del agua, el ir y venir de las mareas, extrayendo su metáfora filosófica central de la Teoría del Caos. – La vida y su imprevisibilidad son amenazadoras, y los problemas humanos cotidianos surgen de las complejas interacciones de las personas con ellas, lo que hace posible el caos.	– «Combatir el estigma y los prejuicios; garantizar el acceso y la calidad de los servicios, ofreciendo una atención integral y una asistencia multiprofesional bajo un enfoque interdisciplinar».

Fuente: elaboración de las autoras.

La dialógica impregna El *Modelo Tidals* hasta el punto de que conceptos que son esencialmente antagónicos por definición se exponen como complementarios. Como en una conexión continua entre opuestos, como la salud y la enfermedad mental, la enfermera y la persona con angustia psicológica, el todo (representado por la persona) y las partes (los dominios del *Yo*, el Mundo y los Otros), aparecen interconectados, caracterizando la enfermería como una actividad interactiva relacionada con el desarrollo y el crecimiento humanos.

Además, el principio rector dEl *Modelo Tidals*, que destaca la crisis como una oportunidad para el cambio, enfrenta a las enfermeras con el enfoque dialógico destacado por Morin, en el que «el ser, organizador de sí mismo, para organizarse, necesita cerrarse al ecosistema, pero también abrirse en relación con él, porque es en este proceso interactivo que se organiza, expresando la relación inseparable entre sujeto y mundo, es decir, el mundo está en el sujeto y el sujeto en el mundo»^(14)^. Por lo tanto, impregnado de complejidad, El *Modelo Tidals* reafirma las relaciones interpersonales como herramienta esencial para el éxito de los cuidados de enfermería en salud mental. Está en consonancia con el paradigma de la atención psicosocial, que recomienda asistir a las personas que sufren enfermedades mentales principalmente en servicios de base territorial y comprender su forma de vida y sus relaciones, permitiéndoles mantener su autonomía y libertad.

La recursividad organizativa está presente en la premisa principal dEl *Modelo Tidals*, según el cual el cambio es constante, y corresponde a la enfermera identificar qué tipo de cambio representa un paso adelante en el proceso de recuperación de la salud mental, desarrollando la conciencia de la persona de que incluso los pequeños pasos son capaces de promover transformaciones. En este punto, está en consonancia con la suposición de Edgar Morin de que todos los resultados de un proceso son también sus coproductores, en un circuito auto constituyente y auto organizador que fomenta acciones dinámicas, interactivas y recursivas, abandonando la concepción lineal de causa y efecto.

La interlocución de teorías refuerza la idea de que el camino hacia la recuperación de la salud mental es único. De esta forma, cada individuo encontrará el mejor camino en el flujo de la vida para alcanzar su bienestar, lo que está en consonancia con la atención humanizada centrada en las necesidades de las personas, proponiendo estrategias de atención diversificadas hacia la integralidad.

El *Modelo Tidals* también expresa la necesidad de comprender a la persona en su totalidad, en tres dominios: el *Yo*, que es el lugar privado de las experiencias humanas, conocido completamente sólo por la persona; el Mundo, en el que la persona comparte algunas experiencias del dominio *Yo*con otros en el mundo social; y los Otros, que es el escenario en el que la persona comparte la vida cotidiana con familiares, amigos, vecinos, colegas de trabajo, profesionales, *etc*., afectándolos y siendo afectada por ellos^(13)^.

Esta premisa corrobora el principio del holograma, que va más allá del reduccionismo, que sólo visualiza las partes, y del holismo, que sólo ve el todo, admitiendo que la parte está en el todo, pero también que el todo está en la parte. Reconoce que es posible aumentar la comprensión de las partes por el todo y del todo por las partes, pasando del pensamiento disyuntivo y reductivo al pensamiento complejo. Contextualizar, por lo tanto, posibilita dar sentido al cuidado humano, permitiendo que florezcan las habilidades necesarias para cuidar de los demás y de la vida, mucho más allá de las habilidades cognitivas, mediante la comprensión de espacios donde todo se construye y reconstruye todo el tiempo, en un proceso permeado por diversas emociones^(15)^. De esta forma, la comprensión humana favorece la armonía entre las personas que son diferentes, ya sea por sus elecciones, origen étnico, orientación sexual o experiencia de sufrimiento, ayudando a allanar el camino para la equidad, considerando los determinantes sociales de la salud, como propone el modelo de atención psicosocial.

Por último, el tetragrama de la complejidad atraviesa El *Modelo Tidals* al afirmar que sólo con la ayuda de la paradoja orden-desorden se puede promover la organización. El concepto de orden va más allá de la idea determinista de estabilidad, apoyándose en la idea de interacciones mutuas, considerando que nada existe sin influencias internas y externas y sin su interdependencia. Además, el desorden abandona la idea de azar, a pesar de admitirla siempre, porque para que haya desorden debe haber «separación, inestabilidades e inconstancias»^(14)^. Por lo tanto, la Teoría de la Complejidad se basa en un pensamiento que considera todas las influencias, tanto internas como externas, destacando la incertidumbre como principio rector, y no propone su eliminación; por el contrario, sugiere que es necesario comprender la contradicción y lo imprevisible a partir de su coexistencia^(14)^.

La integración de estas teorías favorece: la práctica profesional de la enfermera, que implica la personalización de los cuidados, reconociendo la singularidad de cada persona, su historia de vida y su contexto social; la promoción de la autonomía, fomentando la participación del paciente en su proceso de recuperación, valorando su voz y su experiencia; el uso de la flexibilidad y la creatividad para navegar en situaciones de incertidumbre y adaptar sus abordajes; el fortalecimiento de la relación terapéutica, en la que la interacción enfermera-paciente pasa a ser central, promoviendo un vínculo basado en la comprensión y el respeto mutuos.

Así, el pensamiento complejo y El *Modelo Tidals* facilitan el desarrollo de acciones de cuidado en entornos inciertos, como ante las crisis, porque estas acciones se producen analizando conjuntamente las condiciones definidas (orden) e indefinidas (trastorno), y es a partir de esta evaluación que surge un plan de cuidados de salud mental complejo que respeta las historias de vida y los significados de la salud y la enfermedad de las personas en situación de malestar psíquico.

Es necesario reformular el concepto lineal y determinista basado en la certeza para crear modelos que tengan en cuenta la complejidad de la vida. Esto implica adoptar principios teóricos que estén en sintonía con los avances de la enfermería como ciencia, arte y profesión. En este sentido, la complejidad representa un enfoque que enfatiza la inter y transdisciplinariedad como alternativas de reestructuración del conocimiento en la contemporaneidad^(8)^.

El enfoque de la subjetividad necesita aproximar la enfermería a las competencias y habilidades esenciales para la humanización del cuidado, dado que el trabajo clínico y humanizado en salud mental requiere tiempo de acogida, escucha cualificada, atención y relación interpersonal con los sujetos que sufren, con vistas a reorientar el cuidado para centrarse en las posibilidades y potencialidades de los sujetos con vistas a la reinserción social^(16)^.

La teoría de la complejidad se utiliza, en particular, para comprender las relaciones y los fenómenos implicados en los cuidados de enfermería en su conjunto y en las diferentes etapas del desarrollo humano, haciendo hincapié en la necesidad de incorporar múltiples perspectivas para su aplicación^17^. Considerando que la práctica de enfermería se relaciona en gran medida con la complejidad del Ser, las enfermeras, como seres sociales con conocimiento científico técnico, deben reflexionar sobre nuevas formas de cuidado que puedan abarcar el dinamismo y la integralidad, articulando redes de cuidados e interconexiones que dependen de sus acciones^(17)^.

La articulación entre la Teoría de la Complejidad y El *Modelo Tidals*: permite comprender los fenómenos en su complejidad, considerando las interconexiones entre los diferentes elementos (visión sistémica); facilita la adaptación a escenarios en constante cambio y la creación de soluciones más flexibles (adaptabilidad); promueve la colaboración entre diferentes actores y la construcción de soluciones conjuntas (trabajo colaborativo); y contribuye a la construcción de sistemas y organizaciones más resilientes y capaces de hacer frente a la incertidumbre (resiliencia).

Como aplicación práctica a los cuidados clínicos de enfermería en salud mental, cabe destacar: el establecimiento de relaciones interpersonales más significativas, comprendiendo la multiplicidad de factores implicados en la experiencia de sufrimiento de los sujetos, capaces de proporcionar una mayor vinculación de los usuarios y familiares a los servicios y, en consecuencia, un mayor reconocimiento y satisfacción profesional para las enfermeras; la posibilidad de elaborar Proyectos Terapéuticos Singulares más específicos y eficaces, considerando la centralidad de la persona y su historia; y la creación de nuevas estrategias individuales y colectivas de trabajo multidisciplinar de forma integrada, con una mirada ampliada, tan importante para hacer frente a las múltiples demandas que presentan las personas en situación de malestar psíquico.

La interlocución entre la Teoría de la Complejidad y El *Modelo Tidals* permite a las enfermeras desarrollar acciones que colaboren con las premisas de combatir estigmas y prejuicios, garantizar el acceso y la calidad de los servicios, y ofrecer atención integral y asistencia multiprofesional desde una perspectiva interdisciplinaria, fortaleciendo el modelo de atención psicosocial.

## CONCLUSIÓN

Con la intención de consolidar la enfermería en salud mental como una práctica holística, inclusiva, positiva y colaborativa, en detrimento de la enfermería psiquiátrica, paternalista, coercitiva, negativa y orientada a la enfermedad, urge ofrecer una atención clínica en salud mental que supere la visión biologicista, dicotómica y fragmentada, basada en el uso de la medicación, para entender a la persona en malestar psicológico de forma multidimensional, conectando al sujeto con su dimensión sociocultural. Para incentivar esta visión más amplia, se encontraron elementos en la propuesta *del Modelo Tidals* de Phill Barker que dialogan con la Teoría de la Complejidad de Edgar Morin, que está en consonancia con las directrices del modelo de atención psicosocial, presentándose así como una teoría capaz de apuntalar y orientar la práctica de la salud mental y sus complejidades.

Se sugiere que las enfermeras profundicen en el estudio de las teorías enfermeras enfocadas al cuidado de la salud mental, reflexionando críticamente sobre ellas, contribuyendo a la mejora de los modelos, así como adaptando y ampliando su aplicación en diferentes contextos como forma de favorecer su papel como agente terapéutico, colaborando con el rol de los individuos en malestar psicológico y logrando la consolidación de los tan deseados cuidados psicosociales en el sistema sanitario.

El *Modelo Tidals* y la Teoría de la Complejidad, aunque distintos, convergen en sus principios fundamentales para la atención a la salud mental. Al integrar estos conceptos, se crea un marco teórico más robusto que permite comprender al ser humano como un todo, reconociendo la complejidad inherente a los procesos de enfermedad y recuperación en salud mental. Esta reflexión es crucial para superar modelos reduccionistas y fragmentados, promoviendo una práctica de enfermería que valore la subjetividad, la interrelación entre el individuo y el entorno, y la constante transformación presente en la vida humana.
